# Images of Little Cigars and Cigarillos on Instagram Identified by the Hashtag #swisher: Thematic Analysis

**DOI:** 10.2196/jmir.7634

**Published:** 2017-07-14

**Authors:** Jon-Patrick Allem, Patricia Escobedo, Kar-Hai Chu, Tess Boley Cruz, Jennifer B Unger

**Affiliations:** ^1^ Keck School of Medicine University of Southern California Los Angeles, CA United States; ^2^ Center for Research on Media, Technology, and Health University of Pittsburgh Pittsburgh, PA United States

**Keywords:** Instagram, Swisher, little cgars, cigarillos, social media, blunts, health behavior, tobacco use

## Abstract

**Background:**

Little cigar and cigarillo use is becoming more prevalent in the United States and elsewhere, with implications for public health. As little cigar and cigarillo use grows in popularity, big social media data (eg, Instagram, Google Web Search, Twitter) can be used to capture and document the context in which individuals use, and are marketed, these tobacco products. Big social media data may allow people to organically demonstrate how and why they use little cigars and cigarillos, unprimed by a researcher, without instrument bias and at low costs.

**Objective:**

This study characterized Swisher (the most popular brand of cigars in the United States, controlling over 75% of the market share) little cigar- and cigarillo-related posts on Instagram to inform the design of tobacco education campaigns and the development of future tobacco control efforts, and to demonstrate the utility in using big social media data in understanding health behaviors.

**Methods:**

We collected images from Instagram, an image-based social media app allowing users to capture, customize, and post photos on the Internet with over 400 million active users. Inclusion criteria for this study consisted of an Instagram post with the hashtag “#swisher”. We established rules for coding themes of images.

**Results:**

Of 1967 images collected, 486 (24.71%) were marijuana related, 348 (17.69%) were of tobacco products or promotional material, 324 (16.47%) showed individuals smoking, 225 (11.44%) were memes, and 584 (29.69%) were classified as other (eg, selfies, food, sexually explicit images). Of the marijuana-related images, 157/486 (32.3%) contained a Swisher wrapper, indicating that a Swisher product was used in blunt making, which involves hollowing out a cigar and refilling it with marijuana.

**Conclusions:**

Images from Instagram may be used to complement and extend the study of health behaviors including tobacco use. Images may be as valuable as, or more valuable than, words from other social media platforms alone. Posts on Instagram showing Swisher products, including blunt making, could add to the normalization of little cigar and cigarillo use and is an area of future research. Tobacco control researchers should design social media campaigns to combat smoking imagery found on popular sites such as Instagram.

## Introduction

Little cigar and cigarillo use is becoming more prevalent in the United States and elsewhere, with implications for public health [1,2]. These products deliver nicotine to users and share the same health risks as cigarettes [3,4]. However, tobacco control policies in the United States do not apply equally to cigarettes and to little cigars and cigarillos [5]. For example, little cigars and cigarillos can be sold in inexpensive packs of two, whereas cigarettes cannot be legally sold in packages with fewer than 20 cigarettes [6]. Little cigars and cigarillos are also offered in a variety of flavors such as chocolate and strawberry, unlike cigarettes. Lax sales and marketing restrictions could be one reason for the increase in little cigar and cigarillo use in the United States.

As little cigar and cigarillo use grows in popularity, big social media data (eg, Instagram, Google Web Search, Twitter) can be used to capture and document the context in which individuals use, and are marketed, these tobacco products. Big social media data may allow people to organically demonstrate how and why they use little cigars and cigarillos, unprimed by a researcher, without instrument bias and at low costs [7]. These data can be used to inform public policy and pubic health [8], and have been repeatedly used to provide rapid insights into health behaviors [9]. For example, by using these data sources, researchers have shown how hookah use is cross-promoted with alcohol [10], documented reasons for electronic cigarette use [11], and captured reactions to mass media campaigns [12,13]. A study using Twitter data suggested that individuals tend to report smoking specific brands like Swisher when posting about little cigars and cigarillos [14]. A separate study reported that over 80% of little cigar- and cigarillo-related posts on Twitter contained references to marijuana [15].

Instagram is a social media site featuring photo-based content that offers a unique opportunity to examine user-generated images of little cigars and cigarillos. It allows users to capture, customize, and post photos on the Internet. Instagram (with over 700 million active users) [16] has surpassed Twitter’s popularity and is the second most used social media site among youth, behind Facebook [17]. Swisher is the most popular brand of cigars in the United States, controlling over 75% of the market share [18]. In this study, we characterized Swisher little cigar- and cigarillo-related posts on Instagram to inform the design of tobacco education campaigns and the development of future tobacco control efforts, and to further demonstrate the utility in using big social media data in understanding health behaviors.

## Methods

### Data Collection

We collected all data through Instagram’s application programming interface (API). These data were publicly available; that is, anyone with an Internet connection could view the image at the time we retrieved it. Inclusion criteria for this study comprised an Instagram post with the hashtag #swisher. Little cigar and cigarillo products are often referred to by their brand name (eg, Swisher) by users, justifying this inclusion criterion. Participants from focus group studies who regularly used Swisher products revealed that they were unfamiliar with the terms “cigarillos” or “little cigars” [19]. We collected images from Instagram posted between March 6, 2016, and May 21, 2016. This study used a stratified sampling frame based on week, with 11 weeks in the study period, and randomly sampled from each stratum proportionate to the number of posts. A total of 7408 posts included the hashtag #swisher during the study period worldwide, and we randomly sampled 27% of posts each week, yielding 1967 posts to analyze. The authors’ university’s institutional review board approved all study procedures.

### Coding of Themes

Two investigators worked together to become familiar with the data, then generated a coding frame and identified 5 common themes. The purpose of the approach was to condense the raw image-based data into summary format and report the underlying themes that were evident in the data. The primary mutually exclusive themes identified were as follows (1) Tobacco product or promotion: an image of a Swisher product or packaging, professional advertisement, or sponsored flyer, all without the presence of marijuana. (2) Smoking: individual(s) blowing smoke or holding a lit little cigar or cigarillo. (3) Marijuana *:* blunts or hollowed-out cigars next to marijuana on a table, bongs, pipes, joints, rolling papers, or rolled cigars visibly containing marijuana. (4) Meme: a graphic or image that encapsulates a concept, catchphrase, or piece of media [15]. (5) Other: posts that did not clearly fall into one of the above categories (eg, selfies, food, sexually explicit images). We also coded marijuana-themed images for product reference, where a code of 1 indicated that a Swisher wrapper was visible in the image (eg, a wrapper from a Swisher package was next to a hollowed-out cigar) and a code of 0 indicated that a Swisher wrapper was not visible anywhere in the image. In line with prior research using Instagram data to study tobacco-related behavior [10], we determined the presence (coded 1) or absence (coded 0) of alcohol in all images. One investigator coded all posts and another investigator coded a subsample of posts (n=200) to determine reliability. Agreements for the primary themes (90% agreement; κ=.87), product reference (98% agreement; κ=.86), and alcohol (98% agreement; κ=.66) were substantial.

### Descriptive Analysis

We report the percentages of themes and average number of “likes” per theme.

## Results

**Figure 1:**
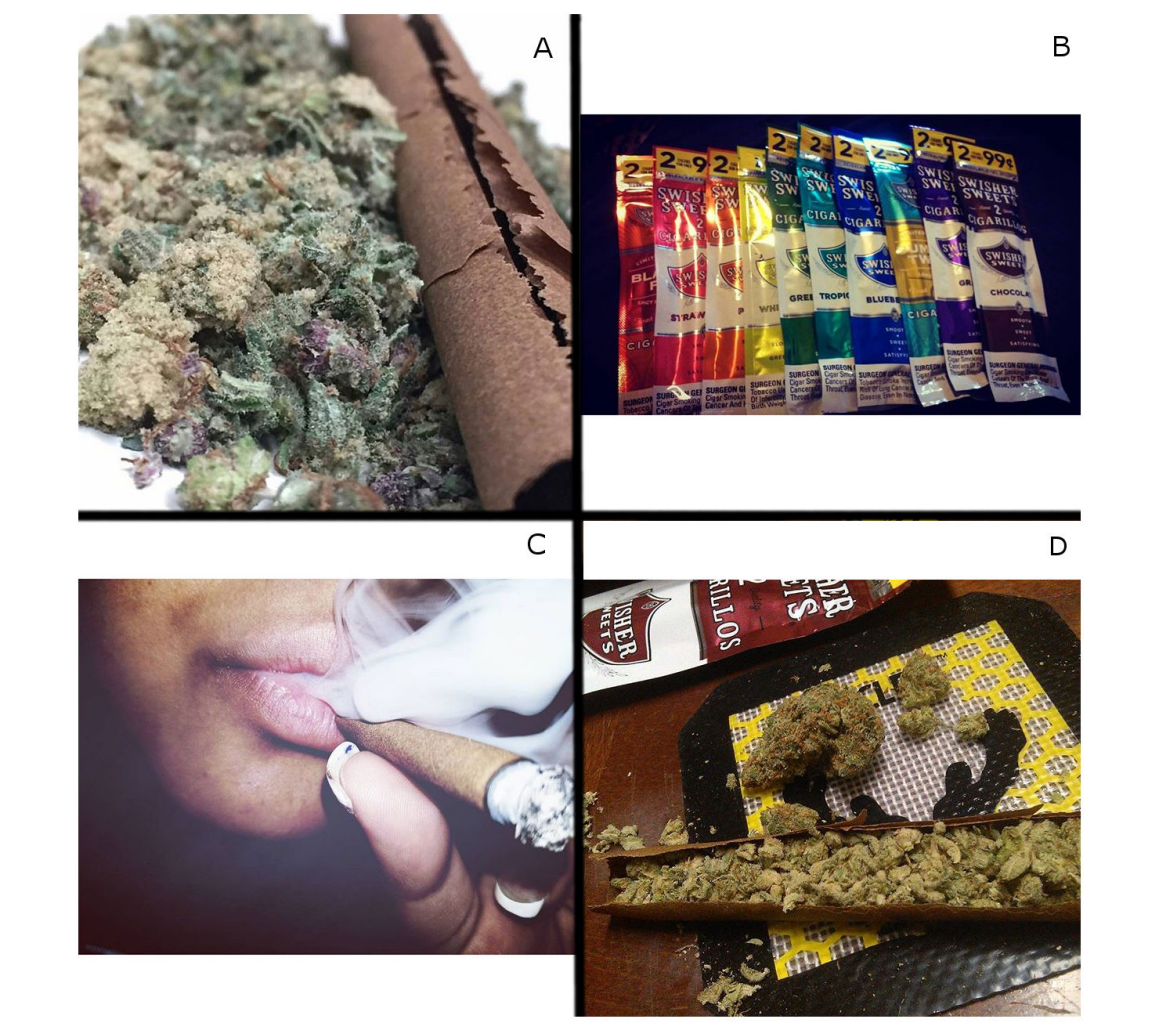
Images representative of themes. (A) marijuana; (B) tobacco product or promotion; (C) smoking; (D) marijuana with product reference.

Among the 1967 posts, 486 (24.71%) were marijuana themed (Figure 1, panel A), 348 (17.69%) were tobacco product or promotion (Figure 1, panel B), 324 (16.47%) showed smoking (Figure 1, panel C), 225 (11.44%) were meme, and 584 (29.69%) were other. Among marijuana images, 157/486 (32.3%) were coded as having a product reference (ie, a Swisher wrapper was visible; Figure 1, panel D). Among all images, 108/1967 (5.49%) showed alcohol. Memes received the highest average number of likes (mean 11, SD 15), followed by other (mean 8, SD 34), marijuana (mean 7, SD 16), smoking (mean 5, SD 9), and tobacco product or promotion (mean 4, SD 7).

## Discussion

### Principal Findings

To our knowledge, this study is the first to use Instagram posts to capture and document the context in which individuals use, and are marketed, little cigars and cigarillos. Images from Instagram may be used to complement and extend the study of health behaviors including tobacco use. Photos may be as valuable as, or more valuable than, words from other social media platforms alone. Posts on Instagram showing Swisher products and smoking imagery, including blunt making, could add to the normalization of little cigar and cigarillo use and should be countered by health campaigns.

We found Swisher products to be used in blunt making, which is similar to earlier reports from focus groups suggesting little cigar and cigarillo companies have developed products that facilitate blunt making [20]. Regular little cigar and cigarillo users reported that in the past, blunt making required a certain level of skill, but in today’s market manufactures have simplified the blunt making process by creating tobacco leaf wraps that come apart easily [20].

Users of blunts may unknowingly expose themselves to nicotine, an issue health campaigns could address on social media. Nicotine is present in the wrapper of a cigar product even if all tobacco filler is removed prior to filling the cigar with marijuana [21]. Consequently, smoking blunts may be considered as concurrent use of marijuana and tobacco [22]. Marijuana use has been associated with substantial adverse effects among youth and young adults, including addiction to other substances, abnormal brain development, progression to use of other drugs, depression or anxiety, motor vehicle accidents, diminished lifetime achievements, and symptoms of chronic bronchitis [23]. Research has suggested that young people recognize blunts as a form of marijuana use but do not recognize it as cigar use [24]. Instagram may be an excellent social media platform to monitor the integration of marijuana with tobacco products, and thus informing new lines of inquiry.

This study found similar results to those in prior studies that used data from Instagram. The tobacco product or promotion theme constituted 17.69% of images in this study, while a previous study on hookah similarly reported that 18% of images showed promotional materials [10]. Among all images in our study, 5.49% showed alcohol, which is less than in prior work, where 31% of all hookah-related images showed or referenced alcohol use [10]. This finding suggests that little cigar and cigarillo use may not co-occur as often as hookah with alcohol consumption.

Photos of individuals smoking constituted 16.47% of the images in this study, while a study on marijuana-related images on Instagram reported that 13% of images pictured people smoking joints or blunts [25]. Similarly, Instagram data on electronic cigarette use or “vaping” showed that 18% of images were of individuals performing tricks with the aerosol or cloud chasing (eg, the act of blowing the largest aerosol cloud possible in a competition) [26]. Positive imagery of tobacco product or marijuana use on social media may lead priority populations such as youth and young adults to view these behaviors as social norms, thus encouraging these behaviors [27]. The impact of viewing smoking-related behaviors online should be studied to identify any possible offline consequences such as uptake of tobacco or increase in its use.

### Limitations

This study relied on Instagram’s API to retrieve data, which prevented access to users with private accounts. This study focused solely on images of posts and not the captions, which may have provided additional insight into little cigar and cigarillo use. Younger age groups (18-29 years) and ethnic minority groups are overrepresented among Instagram users [28]; therefore, these data were not representative of the US population or other populations of interest. However, youth, young adults, and minority groups are priority populations for tobacco-related research, suggesting that Instagram can provide substantial insight into little cigar and cigarillo use. Instagram only allows searches using hashtags, and related posts that do not use the # symbol can be missed. Additionally, Instagram posts with the hashtag #swisher may not represent all posts pertaining to other manufacturers of little cigars and cigarillos.

Despite these limitations, the little cigar- and cigarillo-related themes identified in this study could inform the design of media campaigns that aim to counter the depiction of little cigar and cigarillo use on Instagram, and the development of tobacco control efforts in the future, such as analyzing the potential of new cigar products to be unraveled for use in blunt making. Findings from this study should spur efforts to better understand the consequences of health behaviors viewed on Instagram.

## Abbreviations

API: application programming interface
